# Population Genetics of *Oncomelania hupensis* Snails from New-Emerging Snail Habitats in a Currently *Schistosoma japonicum* Non-Endemic Area

**DOI:** 10.3390/tropicalmed8010042

**Published:** 2023-01-05

**Authors:** Yu-Heng Cheng, Meng-Tao Sun, Ning Wang, Chang-Zhe Gao, Han-Qi Peng, Jie-Ying Zhang, Man-Man Gu, Da-Bing Lu

**Affiliations:** Department of Epidemiology and Statistics, School of Public Health, Soochow University, Suzhou 215131, China

**Keywords:** *Oncomelania hupensis*, new emerging snail habitats, microsatellites, population genetics

## Abstract

Schistosomiasis is still one of the most significant neglected tropical diseases worldwide, and China is endemic for *Schistosoma japonicum*. With its great achievement in schistosomiasis control, the government of China has set the goal to eliminate the parasitic disease at the country level by 2030. However, one major challenge is the remaining huge areas of habitats for the intermediate host *Oncomelania hupensis*. This is further exacerbated by an increasing number of new emerging snail habitats reported each year. Therefore, population genetics on snails in such areas will be useful in evaluation of snail control effect and/or dispersal. We then sampled snails from new emerging habitats in Taicang of Jiangsu, China, a currently *S. japonicum* non-endemic area from 2014 to 2017, and performed population genetic analyses based on nine microsatellites. Results showed that all snail populations had low genetic diversity, and most genetic variations originated from within snail populations. The estimated effective population size for the 2015 population was infinitive. All snails could be separated into two clusters, and further DIYABC analysis revealed that both the 2016 and the 2017 populations may derive from the 2015, indicating that the 2017 population must have been missed in the field survey performed in 2016. These findings may have implications in development of more practical guidelines for snail monitoring and control.

## 1. Introduction

Schistosomiasis is a tropical parasitic disease that has imposed a severe burden on public health and socioeconomic development with over 250 million people currently infected all over the world. China is one of the major endemic countries of schistosomiasis, caused by the zoonotic *Schistosoma japonicum*. In the 1950s, more than 11.6 million were infected and millions of people died of the disease [1]. After decades of integrated multi-disciplinary control efforts in schistosomiasis control, by the end of 2020, the number of infected humans has been reduced to 83,179 [2]. *Oncomelania hupensis* snails are the sole intermediate host of *S. japonicum* and the distribution area of the hosts directly determines the epidemic range of the disease [3]. A recent 2020 trans-country survey reported that out of a total area of 7.37 billion m^2^ investigated for snails, an area of about 2.06 billion m^2^ of snail habitats were recognized, including 1.17 million m^2^ of habitats where snails were detected for the first time (hereinafter referred to as newly emerging snail habitats) [2]. As the risk of schistosomiasis transmission or outbreaks may recur in regions with snails in the context of importing infection reservoirs (e.g., wild rodents [4,5]), snail control is of great importance in schistosomiasis control and final elimination in China [6].

Jiangsu province, China is in the region along the middle and lower reaches of the Yangtze River. The province was once heavily endemic with schistosomiasis japonica. After more than forty years of successful control, the whole region has reached the criteria for schistosomiasis elimination since 1995 [7]. However, there is still a huge area of snail habitats, for example, up to 12.91 million m^2^ snail habitats found during 2011–2018 [8]. Moreover, the number of projects to develop natural wetlands and water resources has recently increased, which may lead to the spread and colonization of snails in new habitats [9,10]. Indeed in 2019 new emerging-snail habitats were reported in one village in Suzhou, Jiangsu [11].

Microsatellites, known as short tandem repeats or simple sequence repeats, are sensitive neutral markers. Due to their ubiquitous occurrence, high copy numbers, heterozygosity and easy detection, as well as inherent potential for variation [12], microsatellites have been applied to population genetic studies of *O. hupensis* snails on the impact of geographical variation, migrant individuals and population diversification [13,14,15]. However, genetic information on snails in newly emerging snail habitats are severely lacking. We have recently used nine polymorphic microsatellites to genotype snails from one new-emerging habitat (in Taicang, Jiangsu) in 2015, and our primary results showed that although the snail population had lower genetic diversity, it had the larger effective population size (Ne) when compared to those from the re-emerging (in Hengtang, Jiangsu) or the previously established and persistent (in Shitai, Anhui) habitats [16]. It would be interesting to know if there is any temporal relationship of snails between and/or among new-emerging habitats in local areas, as in Taicang of Jiangsu new snail habitats were constantly found each year. Such information would be helpful in determining and guiding control measures and strategy for local areas or even for other previously endemic areas where new snail-habitats are frequently found.

## 2. Materials and Methods

### 2.1. Sample Collection and DNA Extraction

**Figure 1:**
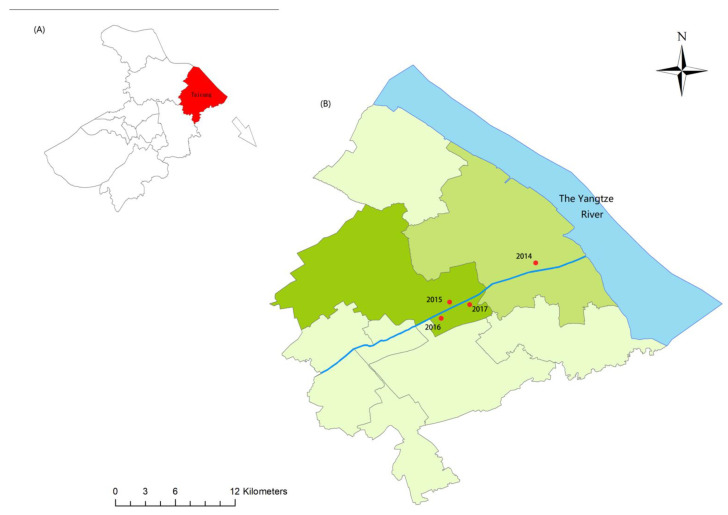
Map for sampling sites. (**A**) Location of Taicang city, Suzhou, Jiangsu of China. (**B**) Four emerging snail habitats along a canal within Taicang city. Red dots, sampled sites.

Annual field surveys for snails, according to the Chinese national monitoring program, had been performed in all suspected habitats in Taicang city of Jiangsu province, China. New-emerged snail habitats were found each year from 2014 to 2017 along a canal, which is connected to the Yangtze River and runs through the Northeast of Taicang (Figure 1 and Table 1). Snail density ranged between 1.60 and 26.26 per frame. We then collected snails from the new snail habitats each year. Although schistosome transmission has been interrupted in the local area since 1995, after which there should be no infected snails with *S. japonicum*, we still checked all sampled snails for schistosome cercariae in the laboratory following standard protocols of cercarial shedding [17]. Adult snails were stored in alcohol before DNA extraction.

### 2.2. DNA Extraction and Microsatellite Genotyping

A total of 157 snails, with 38 from 2014, 40 from 2015, 37 from 2016 and 42 from 2017 (hereinafter referred to as four populations) were sampled for molecular analysis. A small fraction of the head-foot muscle of a snail was obtained for DNA extraction. We used an EZgene^TM^ Mollusc gDNA kit (Biomiga, San Diego, CA, USA) to extract DNA from each snail according to the manufacturer’s guidelines, and then stored it at −25 °C. Snails were genotyped with nine polymorphic microsatellites [16]. The forward primer for each pair was fluorescently labeled with 6-FAM, HEX, TAMRA, or ROX. Two multiplex reactions were performed with the Qiagen Multiplex PCR Kit. Each PCR amplification has a 15μL reaction system, including template DNA 1.5μL, 0.15–0.3 μL of each primer, 7.5 μL Master Mix. RNase-free water was added to the volume. The thermal cycling profile was as follows: 95 °C for 5 min, followed by 30 cycles of 30 s at 94 °C, 60 s at 60 °C, and 30 s at 72 °C, with a final extension at 65 °C for 30 min. The PCR products were sent to Sangong Biotech (Shanghai, China) for genotyping by using an ABI3100 automated sequencer (Applied Biosystems, Foster City CA, USA). Allele sizes were determined using GeneMarker HID (SoftGenetics LLC, State College, PA, USA). 

### 2.3. Genotypic Analysis

#### 2.3.1. Genetic Diversities

Microsatellite data of genotyped snails were exported into an Excel table. The genetic diversity of each population, based on multi-locus, was calculated with GeneAlex 6.5 [18]. The descriptive statistics include number of observed alleles per locus (Na), number of effective alleles per locus (NeA), observed (Ho) and expected heterozygosity (He), unbiased expected heterozygosity (uHe), and inbreeding coefficient (F_IS_).

#### 2.3.2. Genetic Difference and Structure

The analysis of molecular variance (AMOVA) was performed with ARLEQUIN 3.0 [19] to show the hierarchical level of genetic differentiation among and within four populations. The pairwise F_ST_ values and pairwise *Nei* genetic distances were calculated with ARLEQUIN 3.0 and GeneAlex 6.5 to estimate genetic difference between populations.

To construct the genetic substructure of the four snail populations, a Bayesian multi-locus clustering analysis was run with STRUCTURE 2.3 [20] and the most likely number of genetic clusters (K) was estimated. The analysis was based on the ‘admixture’ ancestry model implemented. The burn-in period was set to 10,000 in each run, followed by 10,000 Markov chain Monte Carlo (MCMC) iterations. Independent runs were performed with the values of K from one to six, and each K was repeated 20 times to check the consistency of the results. To identify the optimal value of K, we calculated the statistic Delta K (ΔK), which is based on the change rate in the calculated log probability between successive K values [21]. The online program STRUCTURE HARVESTER was used to analyze ΔK and then to estimate the number of subpopulations according to the peak of the ΔK graph [22]. CLUMPP 1.1 [23] was used to average ancestry coefficients of snail individuals across independent runs. Distruct 1.1 was used to visualize clusters inferred [24]. A principal coordinate analysis was performed with GeneAlex 6.5 to investigate the patterns of genetic relationships between snail individuals. A cluster analysis was run with MEGA 11 [25], based on Unweighted Pair Group Method with Arithmetic Mean (UPGMA) algorithm and a phylogenetic tree was plotted to show the genetic association among snail populations.

#### 2.3.3. Effective Population Size

To estimate the effective population size (i.e., Ne, referring to the number of parental individuals who could contribute to the next generation effectively [26]), we used two software packages, NeEstimator 2.1 (NeEst) [27] and LDNe [28], to calculate Ne values and their 95% confidence intervals (CIs) for each population. In both software packages, the calculation was based on linkage disequilibrium. In estimation progress, alleles with a frequency above or equal to 0.05 were included.

#### 2.3.4. Population Divergence History

The Approximate Bayesian Computation method was run with DIYABC [29] to analyze the demographic history of the four snail populations and then to infer their historical migration path [30,31]. Based on the sampling time schedule (i.e., from 2014 to 2017 with one snail population from each year) and the location of sampling sites (Figure 1), we defined 33 possible scenarios with the 2014 population as the first (i.e., the common ancestor) (Supplementary Table S1), taking both admixture and no admixture into consideration [32]. The definitions of different parameters and their prior distributions were provided in Supplementary Table S2. For microsatellite data in analyses we employed the most appropriate mutational model—the generalized stepwise mutation model, which could decrease the number of parameters [33]. In a scenario (Supplementary Table S2), we defined t2 > t1 and t3 > t2 and chose mean allele number, mean genetic diversity, F_ST_, and other indicators as summary statistics, and each generated 1,000,000 simulated data sets. Following that, we calculated the Euclidian distance between the observed data set and each simulated data set, which had previously been standardized. We calculated the posterior probability for each scenario and its 95% CIs through a local linear regression procedure [34,35]. The best scenario was chosen and a principal component analysis (PCA) was performed to examine fitness between the simulated and the observed.

## 3. Results

### 3.1. Genetic Diversity

As seen in Table 2, among four snail populations Na (no. of different alleles per locus) ranged from 3.333 to 4.889, and NeA (no. of effective alleles per locus) varied between 2.172 and 2.682. The observed heterozygosity ranged from 0.343 to 0.391, and the expected heterozygosity varied between 0.447 and 0.538. The values of He, including the unbiased expected heterozygosity (uHe), in each of the four snail populations were higher than their corresponding values of Ho, and all F_IS_ values were greater than zero, indicating a loss of heterozygosity.

### 3.2. Genetic Difference and Structure

The AMOVA displayed that the variations mainly existed within snail populations, which accounted for 81.03 % of the total variations, and among populations were 18.97% (Table 3). The pairwise genetic distances between four populations showed that the 2015 and the 2016 were closest at 0.193, while the 2014 and the 2017 were most distant at 2.394. The pairwise F_ST_ values ranged from 0.066 to 0.320 with all *p* values <0.05. The 2014 population was clearly separated from all other three populations (Table 4).

**Figure 2:**
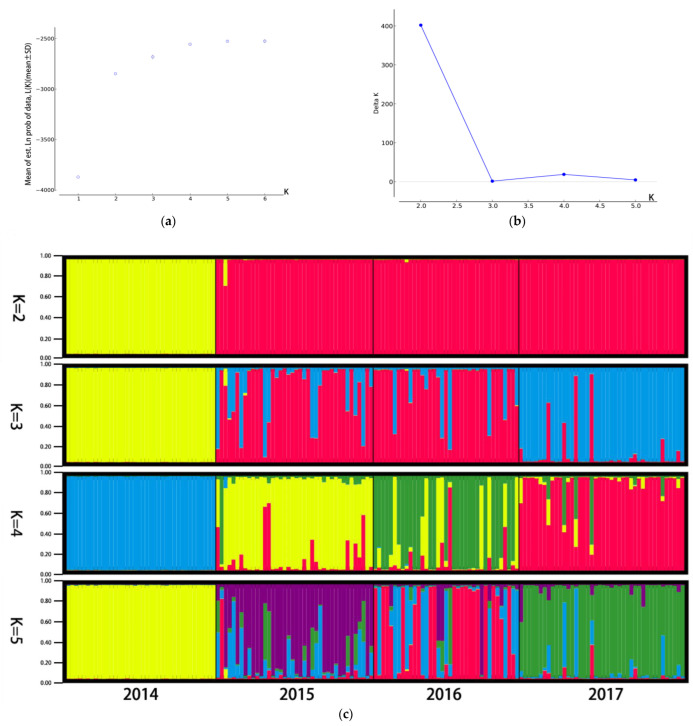
Population structure of four snail populations. (**a**) Mean (±SD) natural logarithm of the likelihood of the data [LnP(X|K)] for each value of assumed clusters (K). (**b**) Delta K values are plotted against the number of assumed K. (**c**) Genetic structure with the K value, respectively, taking 2, 3, 4 and 5. Each snail individual is represented by a thin vertical line, which is partitioned into K coloured segments that represents the individual’s estimated membership fractions in K clusters. The figure shown for a given K is based on the highest probability run at that K. Snail populations are labelled below the figure. Correspondence of colours across figures is not meaningful.

**Figure 3:**
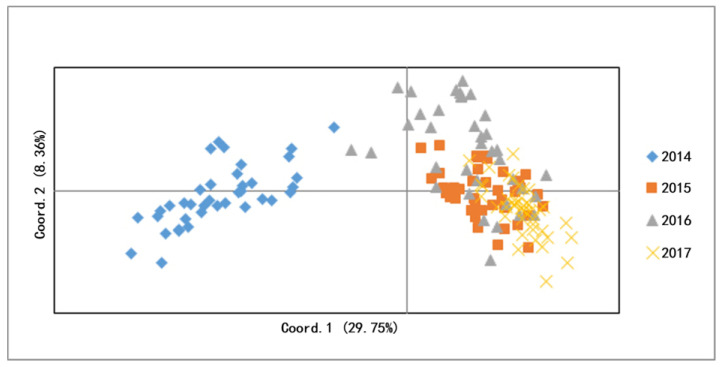
PCOA of four snail populations.

**Figure 4:**
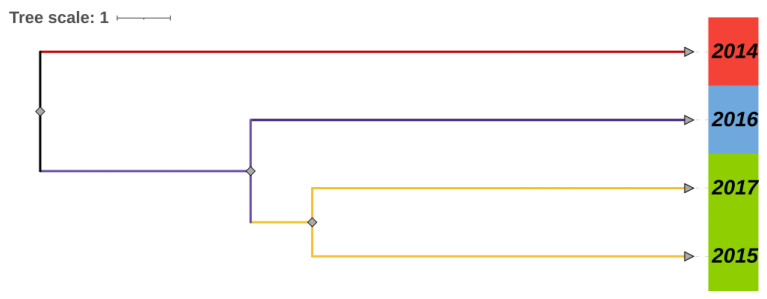
UPGMA tree of four snail populations.

From the STRUCTURE software carried out with Bayesian analysis, the highest likelihood was obtained when K equals two, suggesting that all snails from four populations can be assigned into two clusters (Figure 2). The PCOA plot from correspondence analysis based on the covariance of the genetic distance matrix showed the same results (Figure 3). With the UPGMA cluster analysis, the 2015 population first clustered with the 2017 and then with the 2016, and the formed branch was finally separated from the 2014 population (Figure 4).

### 3.3. Effective Population Size (Ne)

Both NeEstimator and LDNe, based on linkage disequilibrium, revealed comparable and congruent estimates of Ne (being infinitive) for the 2015 population. Among the other three populations, the point estimates of Ne varied greatly (from 20.7 in 2016 to 3687.7 in 2017) with NeEst, but not with LDNe (from 43.0 in 2014 to 53.9 in 2016) (Table 5).

### 3.4. Population Divergence History

**Figure 5:**
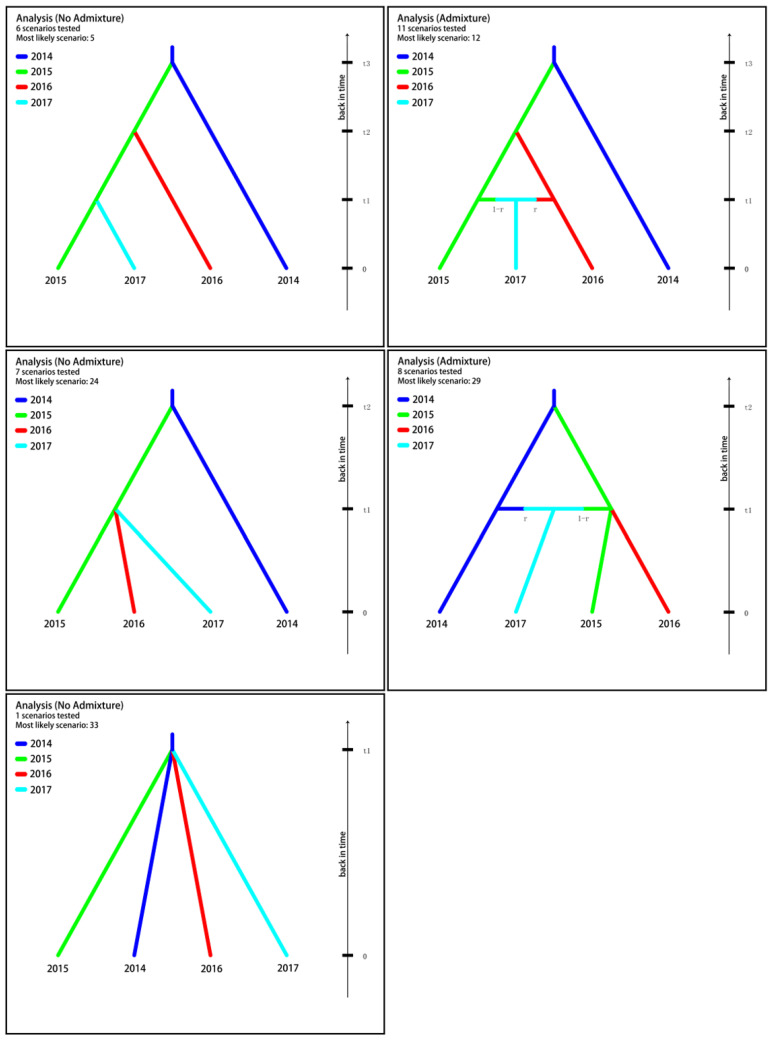
Graphic representation of the five most likely scenarios inferred in DIYABC analysis.

**Figure 6:**
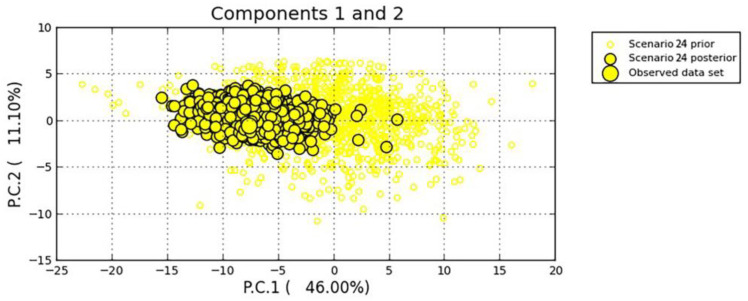
Goodness fit of Scenario 24 to the observed data evaluated in PCA analysis.

With DIYABC analysis the posterior probability for each of the assumed 33 scenarios was calculated and presented in Supplementary Table S3. From the first round of the selection process, the five best scenarios (i.e., Scenario 5, 12, 24, 29 and 33) were obtained, from which Scenario 24 with the highest posterior probability was selected after the second round. See Figure 5 and Table 6. In Scenario 24, the 2015 population first split from the 2014, then migrated and diffused, and finally formed two populations—the 2016 and the 2017. The good fit between the scenario and the observed data was presented in Figure 6, a PCA analysis based on 10,000 simulations.

## 4. Discussion

In this study, the genetic diversities (i.e., Na, He, and so on) of snails in new-emerged habitats were generally lower when compared to those in the previously established and persistent snail habitat [13,16]. For example, Guan et al. [13] reported that within the same province the values of Na and He in Yangzhong were, respectively, 13.750 and 0.901, and in Yizheng 11.875 and 0.910, both considerably higher than those observed in our study. Low genetic diversity could be associated with lower fitness of hosts and possible facilitation of parasitism at the level of populations [36,37]. The causes of low diversity may be related to high degrees of habitat disturbance, small population sizes, or recently established sites. For example, in Senegal, which is endemic for *Schistosoma mansoni*, population genetic perturbations resulted in a reduced intrapopulation diversity [9]. We predicted that the observed low diversity here may be mainly due to one or a combination of the factors mentioned above, as local snail control with a focus on molluscides has been performed once snails are found.

The genetic difference (F_ST_) observed among the four snail populations was from low (i.e., 0.066) to great (0.320), based on Wright’s criterion [38]. The highest was between the 2014 and the 2017, and most of the genetic variation was from within snail populations. It would then be predicted that some snail populations or individuals may have the potential for propagation and perhaps be more susceptible to schistosomes if exposed [39,40]. Compared to the other three populations, the 2015 population showed the greatest effective population size with both NeEstimator and LDNe software. This suggested that this population could be the most adaptable to the environment and have reproductive potential, leading to the possibility for migration and diffusion and then colonizing of new habitats. The further evidence of this was provided from DIYABC analyses. Among a total of 33 scenarios illustrating a possible relationship among/between four snail populations, the best scenario with the highest posterior probability (i.e., 95.72% in the first round among 33 scenarios, and 53.26% in the second round among five scenarios) was identified, in which the 2015 population very likely derived from the 2014 and then spread and separately formed the 2016 and the 2017 populations. Results from STRUCTURE, and partly from phylogeny analysis, showed that four snail populations were grouped into two clusters, with three populations (the 2015, the 2016 and the 2017) into one cluster and the 2014 into another, suggesting a different demographical history among four snail populations. This is consistent with the above results. We therefore raised the hypothesis that due to an annual flooding occurring along the Yangtze River, coupled with the movement ecology of *O. hupensis*, the 2014 snail population in the new emerging habitat might come from its upper reaches nearby, as Taicang of Jiangsu is connected via several canals to the river and snail dispersal may occur along the waterways [41,42,43]. The 2015 population, which might be descended from the 2014, may have spread and then form the 2016 and the 2017, the latter of which might have been missed in the local snail survey performed in 2016. Snails have been frequently overlooked in the field survey, as the current boots-on-the ground approach for snail surveys in the field is both laborious and inefficient [44].

Elimination of a human parasitic disease such as schistosomiasis japonica is challenging, as *S. japonicum* can persist in a range of nonhuman mammalian hosts, including over 40 mammal species. As control strategies for the parasite infection in wild animals (e.g., rodents) may not prove logistically feasible or practical, control and removal of the intermediate host snails may be an alternative with the ongoing focus on molluscides and/or environment management. Therefore, our understanding of the genetic characteristics and dispersal potential of *O. hupensis* in new emerging snail habitats in Taicang of Jiangsu, China may provide a basis for formulating targeted prevention measures, which would exert epidemiological implications for final schistosomiasis elimination across the country [45,46].

## 5. Conclusions

This study, based on microsatellite analyses, demonstrated that in newly emerging snail habitats in Taicang of Jiangsu, China, all snail populations (from 2014 to 2017) generally had low genetic diversity. The estimated effective population size for the 2015-population was infinitive, whereas those for all other three populations did not vary greatly when computed with LDNe. Four populations of snails could be separated into two clusters, and further DIYABC analysis revealed that both the 2016 and the 2017 populations derived from the 2015, indicating that the 2017 population must have been missed in the field survey performed in 2016.

Our study sampled snails from newly emerged snail habitats within a canal in Taicang of Jiangsu, China. The results of population genetic analyses with microsatellites showed a low genetic diversity in all snail populations when compared to persistent populations. For the first time, we did provide genetic evidence that snails had been overlooked in annual field surveys. These findings may have implications in the development of more practical guidelines for local snail monitoring and control.

## Figures and Tables

**Table 1:** Information on sampling sites.

Site/Year	Snail Habitat (m^2^)	No. Surveyed Frames (/0.11 m^2^)	No. Snail Frames (/0.11 m^2^)	Snail Density (/0.11 m^2^)
2014	1425	250	5	1.60
2015	600	32	26	6.88
2016	700	30	24	7.03
2017	1330	25	27	26.26

**Table 2:** Genetic diversity of each snail population.

Population		N	Na	NeA	Ho	He	uHe	F_IS_
2014	Mean	30.778	3.333	2.568	0.349	0.536	0.544	0.377
	SE	3.957	0.601	0.330	0.088	0.079	0.080	0.110
2015	Mean	33.778	4.556	2.682	0.391	0.503	0.509	0.301
	SE	4.037	0.959	0.436	0.132	0.103	0.104	0.161
2016	Mean	28.333	4.889	2.654	0.343	0.538	0.551	0.495
	SE	3.686	0.790	0.394	0.101	0.078	0.078	0.130
2017	Mean	37.778	3.667	2.172	0.384	0.447	0.452	0.138
	SE	3.639	0.601	0.303	0.089	0.090	0.092	0.088

N, number of snails with loci successfully genotyped. Na, no. of different alleles per locus. NeA, no. of effective alleles per locus. Ho, observed heterozygosity. He, expected heterozygosity. uHe, unbiased expected heterozygosity. F_IS_, the inbreeding coefficient.

**Table 3:** Analysis of molecular variance (AMOVA) of four snail populations.

Source of Variation	Sum of Squares	Variance Components	Percentage of Variation	Fixation Index
Among populations	82.084	0.331	18.97	F_ST_ = 0.190 *
within populations	438.184	1.414	81.03	

* *p* < 0.05.

**Table 4:** Pairwise genetic distance (above) and F_ST_ values (below) of four populations.

Population	2014	2015	2016	2017
2014		2.185	2.064	2.394
2015	0.241 *		0.193	0.196
2016	0.236 *	0.066 *		0.264
2017	0.320 *	0.094 *	0.133 *	

* *p* < 0.05.

**Table 5:** Effective population size (Ne) and 95% CIs based on Linkage disequilibrium.

Population	With NeEst	With LDNe
2014	175.6 (11.5, 898.5)	43.0 (16.2, Inf)
2015	Inf (33.0, Inf)	Inf (69.8, Inf)
2016	20.7 (4.8, 31.6)	53.9 (17.4, Inf)
2017	3687.7 (15.2, Inf)	52.0 (20.0, Inf)

Inf, infinite.

**Table 6:** Posterior probabilities of the five best scenarios inferred in DIYABC Analysis.

Analysis	Scenario Code	Scenario Profile			Posterior Probability	95% CI
		t3	t2	t1		
No admixture (t1 t2 t3)	5	2014 → 2015	2015 → 2016	2015 → 2017	0.2561	[0.2376,0.2747]
Admixture (t1 t2 t3)	12	2014 → 2015	2015 → 2016	2015 + 2016 → 2017	0.1964	[0.1811,0.2116]
No admixture (t1 t2)	24		2014 → 2015	2015 → 2016; 2015 → 2017	**0.5326**	**[0.5114,0.5538]**
Admixture (t1 t2)	29		2014 → 2015	2015 → 2016; 2014 + 2015 → 2017	0.0138	[0.0045,0.0230]
No admixture (t1)	33			2014 → 2015; 2014 → 2016; 2014 → 2017	0.0011	[0.0000,0.0102]

## Data Availability

Not applicable.

## Supplementary Materials

The following supporting information can be downloaded at: https://www.mdpi.com/article/10.3390/tropicalmed8010042/s1, Table S1: Parameters and prior distributions used in DIYABC analysis; Table S2: The assumed 33 demographic history scenarios among four snail populations; Table S3: Posterior probabilities of 33 demographic history scenarios evaluated in DIYABC analysis; Table S4: Posterior distributions of population demographic parameters from Scenario 24 inferred in DIYABC analysis.
