# Supplementary material for: Population Genetics of Oncomelania hupensis Snails from New-Emerging Snail Habitats in a Currently Schistosoma japonicum Non-Endemic Area
Source: Trop Med Infect Dis. 2023 Jan 5;8(1):42. doi: 10.3390/tropicalmed8010042 (PMC9861412; doi:10.3390/tropicalmed8010042)
Supplement: Supplementary file 1 [file tropicalmed-08-00042-s001.zip › tropicalmed-2095563-supplementary.pdf]

Table S1. Definitions of different parameters and prior distributions with DIYABC.

Parameter	Distribution	Interval
Effective population size, Ni	Uniform	10–10,000
Rates of admixture, ra	Uniform	0.001–0.999
Mean mutation rate (MEANMU)	Uniform	10^{-4}–10^{-3}
Mean parameter of the geometric distribution of the number of repeats (MEAN P)	Uniform	0.1–0.3
Mean single nucleotide insertion/deletion mutation rate (MEANSNI)	Log-uniform	10^{-8}–10^{-5}

Table S2. Assumed 33 demographic history scenarios among four snail populations.

Analysis	Number	Scenario		
		t3	t2	t1
No admixture（t1 t2 t3)	1	2014→2015	2014→2016	2014→2017
	2	2014→2015	2014→2016	2015→2017
	3	2014→2015	2014→2016	2016→2017
	4	2014→2015	2015→2016	2014→2017
	**5**	**2014→2015**	**2015→2016**	**2015→2017**
	6	2014→2015	2015→2016	2016→2017
Admixture（t1 t2 t3)	7	2014→2015	2014→2016	2014 + 2015→2017
	8	2014→2015	2014→2016	2014 + 2016→2017
	9	2014→2015	2014→2016	2015 + 2016→2017
	10	2014→2015	2015→2016	2014 + 2015→2017
	11	2014→2015	2015→2016	2014 + 2016→2017
	**12**	**2014→2015**	**2015→2016**	**2015 + 2016→2017**
	13	2014→2015	2014 + 2015→2016	2014→2017
	14	2014→2015	2014 + 2015→2016	2015→2017
	15	2014→2015	2014 + 2015→2016	2016→2017
	16	2014→2015	2014 + 2015→2016	2014 + 2016→2017
	17	2014→2015	2014 + 2015→2016	2015 + 2016→2017
No admixture（t1 t2)	18		2014→2015; 2014→2016	2014→2017

	19	2014→2015; 2014→2016	2015→2017
	20	2014→2015; 2014→2016	2016→2017
	21	2014→2015	2014→2016; 2014→2017
	22	2014→2015	2015→2016; 2014→2017
	23	2014→2015	2014→2016; 2015→2017
	24	2014→2015	2015→2016; 2015→2017
Admixture（t1 t2)	25	2014→2015; 2014→2016	2014 + 2015→2017
	26	2014→2015; 2014→2016	2015 + 2016→2017
	27	2014→2015; 2014→2016	2014 + 2016→2017
	28	2014→2015	2014→2016; 2014 + 2015→2017
	**29**	2014→2015	2015→2016; 2014 + 2015→2017
	30	2014→2015	2014 + 2015→2016； 2014→2017
	31	2014→2015	2014 + 2015→2016； 2015→2017
	32	2014→2015	2014 + 2015→2016； 2014 + 2015→2017
No admixture(t1)	33		2014→2015； 2014→2016； 2014→2017

Table S3. Posterior probabilities of the 33 demographic history scenarios evaluated in DIYABC analysis.

Analysis	Number	Posterior Probability	95% CI
No admixture（t1 t2 t3）	1	0.0001	[0.0000,0.0033]
	2	0.0096	[0.0052,0.0140]
	3	0.0026	[0.0000,0.0058]
	4	0.0003	[0.0000,0.0035]
	**5**	**0.8506**	**[0.8298,0.8714]**
	6	0.1368	[0.1164,0.1572]
Admixture（t1 t2 t3）	7	0.0001	[0.0000,0.0106]
	8	0.0002	[0.0000,0.0106]
	9	0.0034	[0.0000,0.0138]
	10	0.0208	[0.0095,0.0320]
	11	0.0111	[0.0003,0.0218]
	**12**	**0.7133**	**[0.6705,0.7560]**
	13	0.0000	[0.0000,0.0105]
	14	0.1990	[0.1579,0.2402]
	15	0.0140	[0.0032,0.0248]
	16	0.0016	[0.0000,0.0120]
	17	0.0365	[0.0241,0.0488]
No admixture（t1 t2）	18	0.0008	[0.0003,0.0013]

	19	0.0275	[0.0201,0.0348]
	20	0.0083	[0.0056,0.0111]
	21	0.0025	[0.0009,0.0040]
	22	0.0014	[0.0007,0.0022]
	23	0.0023	[0.0010,0.0036]
	**24**	**0.9572**	**[0.9483,0.9661]**
Admixture（t1 t2 ）	25	0.0047	[0.0000,0.0524]
	26	0.0456	[0.0000,0.0915]
	27	0.0035	[0.0000,0.0512]
	28	0.0005	[0.0000,0.0486]
	**29**	**0.4901**	**[0.4404,0.5397]**
	30	0.0005	[0.0000,0.0486]
	31	0.4363	[0.3848,0.4879]
	32	0.0188	[0.0000,0.0656]
No admixture(t1)	33		
**The second round**			
No admixture VS Admixture	5	0.2561	[0.2376,0.2747]
	12	0.1964	[0.1811,0.2116]
	**24**	**0.5326**	**[0.5114,0.5538]**
	29	0.0138	[0.0045,0.0230]
	33	0.0011	[0.0000,0.0102]

Table S4. Posterior distributions of population demographic parameters from Scenario 24 with the highest posterior probability inferred by DIY ABC analysis.

Parameter	Mean	Median	Mode	Quantile 2.50%	Quantile 5.00%	Quantile 95.00%	Quantile 97.5%
N1	2.38×10^3	2.13×10^3	1.82×10^3	6.94×10^2	8.55×10^2	4.70×10^3	5.88×10^3
N2	3.85×10^3	3.59×10^3	3.29×10^3	1.19×10^3	1.47×10^3	7.33×10^3	8.37×10^3
N3	6.21×10^3	6.29×10^3	7.02×10^3	2.21×10^3	2.79×10^3	9.41×10^3	9.71×10^3
N4	2.76×10^3	2.31×10^3	1.44×10^3	5.46×10^2	6.95×10^2	6.69×10^3	7.85×10^3
t1	8.04×10^2	6.16×10^2	3.32×10^2	1.04×10^2	8.95×10^1	2.08×10^3	2.78×10^3
t2	8.09×10^3	8.42×10^3	9.75×10^2	4.51×10^2	5.10×10^2	9.86×10^3	9.93×10^3
µmic	2.28×10^{-4}	1.91×10^{-4}	1.43×10^{-4}	1.09×10^{-4}	1.15×10^{-4}	4.72×10^{-4}	5.89×10^{-4}
pmic	1.90×10^{-1}	1.85×10^{-1}	1.49×10^{-1}	1.06×10^{-1}	1.11×10^{-1}	2.82×10^{-1}	2.90×10^{-1}
snimic	5.25×10^{-6}	5.47×10^{-6}	1.00×10^{-5}	8.36×10^{-8}	1.57×10^{-7}	1.00×10^{-5}	1.00×10^{-5}

N1, N2, N3 and N4, effective population sizes of the corresponding populations;
t1, divergence of 2016 and 2017 from 2015; t2, divergence of 2016 from 2015;
µmic, the mean mutation rate of SSR;
pmic, the mean distribution of the number of repeats of microsatellites;
snimic, the mean rate of single nucleotide insertions/deletions.
